# Impact of social media addiction on college students’ academic procrastination: a chain mediated effect of lack of self-control and fear of missing out

**DOI:** 10.3389/fpsyg.2025.1668567

**Published:** 2025-10-20

**Authors:** Yuxi Tang, Weiguang He

**Affiliations:** College of Social Sciences, Shenzhen University, Shenzhen, China

**Keywords:** social media addiction, academic procrastination, lack of self-control, fear of missing out, chain mediated effect

## Abstract

**Aim:**

Social media addiction is increasingly receiving global attention, and may exacerbate the mental health problems and academic procrastination in college students. However, the complex relationship and underlying mechanisms between social media addiction and academic procrastination remain unclear. This study aimed to explore how social media addiction affects academic procrastination in college students, and to identify the chain mediating role played by lack of self-control and fear of missing out in this relationship, toward the development of effective interventions for mental health and learning performance.

**Method:**

This cross-sectional survey study recruited 825 college students from 30 provincial-level administrative regions in China through online platforms in June 2025. Four mature scales were used to measure social media addiction, academic procrastination, lack of self-control, and fear of missing out, and Bootstrap method was used to test the mediating effect hypothesis.

**Result:**

Social media addiction, fear of missing out, and lack of self-control positively predicted college students’ academic procrastination. Lack of self-control and fear of missing out not only played separate mediating roles between social media addiction and academic procrastination, but also jointly constituted a chain mediation between them.

**Discussion:**

This study expands the research on the relationship between social media addiction and academic procrastination, providing new insights into reducing the negative impact of social media addiction in the digital age, improving college students’ academic performance, and promoting their mental health.

## 1 Introduction

At present, college students frequently use various types of social media, which has a profound impact on their mental health and academic performance (Nguyen et al., 2025), and can easily lead to social media addiction problems (Aslan and Yasar, 2020; Lin et al., 2023; Piko et al., 2024), which has caused strong concerns among educators and policymakers. At the same time, academic procrastination among college students is becoming a global phenomenon (Gareau et al., 2019; Peixoto et al., 2021; Gadosey et al., 2024). Previous studies have found a correlation between academic procrastination and social media addiction (Kurker and Surucu, 2024). However, the complex relationship between social media addiction and academic procrastination among college students has not been fully elucidated, and further research in this field can provide a basis for developing effective intervention measures.

### 1.1 Social media addiction and academic procrastination

Social media addiction refers to individuals’ difficulty in effectively controlling their excessive use of various social media behaviors (Blackwell et al., 2017; D'Arienzo et al., 2019). Some college students may be attracted by various attractive information services and entertainment activities on social media (Gulnar, 2025; Salari et al., 2025), leading to excessive use and dependence on social media. This social media addiction may disrupt their normal learning, causing them to lose interest in their studies and find it difficult to complete academic tasks on time. Academic procrastination refers to the phenomenon where learners are unable to complete academic tasks within a specified time frame, which may be due to improper time management, poor execution ability, lack of rational awareness, lack of sense of responsibility, and fear of failure (Rabin et al., 2011; Steel and Klingsieck, 2016; Peixoto et al., 2021). Academic procrastination is a common academic and psychological problem among college students (Gershoni and Stryjan, 2023). In the digital age, college students may experience academic procrastination due to excessive addiction to online games or social media (Parmaksiz, 2023). Previous studies have suggested a possible correlation between social media addiction and academic procrastination (Liu et al., 2022; Caratiquit and Caratiquit, 2023). However, there are still differences in research on the influencing factors and specific pathways of the relationship between social media addiction and academic procrastination, and the exploration of the complex mediating mechanisms is not deep enough. This study attempts to explore how social media addiction affects academic procrastination through multiple pathways through a chain mediation model. Social media addiction may deplete the self-control resources of college students, weaken their willpower, erode their perseverance in dealing with learning challenges, increase their anxiety and stress, and make them easily avoid learning difficulties (Ning and Inan, 2024), which may lead to academic procrastination. Therefore, we propose the following hypothesis:

*H1*: The degree of social media addiction can positively predict academic procrastination among college students.

### 1.2 Lack of self-control as a potential mediator in the relationship between social media addiction and academic procrastination

Self control refers to an individual’s ability to effectively overcome interference, resist temptation, and control their behavior in daily life to achieve expected goals (Baumeister et al., 2007; Uziel and Baumeister, 2017; Grund and Carstens, 2019). For college students, the lack of self-control is an important factor that not only makes it difficult for them to reduce external interference but also leads to poor academic performance (King and Gaerlan, 2014). Previous studies have shown a correlation between social media addiction and self-control ability (Koç et al., 2023; Garcia and Cooper, 2024). The widespread phenomenon of social media addiction among college students indicates that they have difficulty effectively regulating and controlling excessive social media use. Long term social media addiction may weaken students’ self-control ability (Blachnio et al., 2023). In addition, students who lack self-control may be less willing to take action when facing academic difficulties, and may have difficulty effectively controlling their emotions or mobilizing willpower to align their actions with their goals (Dunbar et al., 2018), which may lead to academic procrastination. The self-control theory holds that human control resources are limited, and some behaviors that require self-control to regulate will consume self-control resources, thereby hindering the completion of subsequent tasks (Jedrzejczyk and Zajenkowski, 2020). This means that social media addiction may consume a significant amount of control resources for college students, affecting the timely completion of academic tasks. Therefore, a second hypothesis is proposed:

*H2*: College students’ lack of self-control plays a mediating role between their social media addiction and academic procrastination.

### 1.3 Fear of missing out (FOMO) is a potential mediator in the relationship between social media addiction and academic procrastination

Fear of missing out (FOMO) refers to the anxiety caused by the fear of missing out on social activities, experiences, or information to participate in activities with friends (Przybylski et al., 2013; Beyens et al., 2016; Milyavskaya et al., 2018; Elhai et al., 2021). College students are usually in a critical period of building good social relationships and tend to pay more attention to and actively participate in their friends’ social activities. Missing certain social activities may trigger social anxiety (Sabir and Jabeen, 2023). Social media can exacerbate FOMO to some extent, and when college students learn about their friends’ rich social lives through social media, it may exacerbate their anxiety and unease about missing out (Yüksel et al., 2025). Previous studies have shown a correlation between social media addiction and FOMO (Blackwell et al., 2017; Zhang et al., 2021; Aygar et al., 2025), but these studies have not yet clearly and comprehensively revealed the relationship between social media addiction, FOMO, and academic procrastination. The theory of FOMO suggests that people’s FOMO involves two core processes: first, the perception of missing important social experiences; second, individuals forcing themselves to be more actively involved in social activities to alleviate this anxiety (Gupta and Sharma, 2021). College students with social media addiction tend to pay more attention to their friends’ social activities or experiences, which may exacerbate their FOMO (Zhao, 2024). This social anxiety may force them to spend more time on social media, hindering their ability to complete academic tasks on time (Al-Furaih and Al-Awidi, 2021). In addition to spending more time and energy on social media, college students with social media addiction may experience higher FOMO, which may lead to academic procrastination (Li and Ye, 2022). Therefore, the following hypotheses are proposed:

*H3*: College students’ FOMO plays a mediating role between their social media addiction and academic procrastination.

### 1.4 Lack of self-control and FOMO are potential chain mediators in the relationship between social media addiction and academic procrastination

Previous studies have shown a correlation between social media addiction and FOMO (Sun et al., 2022; Jiao and Cui, 2024), and a close relationship between FOMO and lack of self-control (Kumar and Kumar, 2025). However, previous studies have not yet integrated and analyzed the relationship between social media addiction, academic procrastination, self-control, and FOMO, thus failing to clearly reveal the complex mechanisms by which social media addiction affects academic procrastination. According to the theory of self-control, social media addiction can deplete the self-control resources of college students (Hagger et al., 2010), making it difficult for them to regulate their excessive social media use behavior. This may exacerbate their FOMO (Coker et al., 2025). The FOMO is associated with students’ negative social psychological traits, which can have a negative impact on their academic performance (Milyavskaya et al., 2018; Al-Busaidi et al., 2023), thereby making them more likely to procrastinate academically. Therefore, there is reason to believe that college students’ dependence on social media may weaken their self-control ability, thereby affecting their FOMO and exacerbating academic procrastination, ultimately forming a complete chain mediation mechanism. In response to this situation, the following hypotheses have been proposed:

*H4*: College students’ lack of self-control and FOMO play a chain mediating role in the relationship between their social media addiction and academic procrastination.

**Figure 1:**
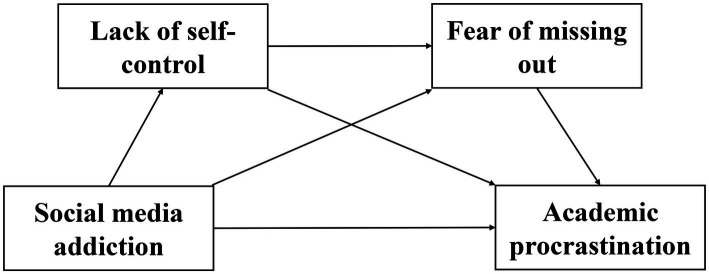
Chain mediation model between social media addiction and academic procrastination among college students.

Although there have been a few studies exploring the mediating mechanisms between social media addiction and academic procrastination, there has been no systematic exploration of the interrelationships among social media addiction, academic procrastination, lack of self-control, and FOMO from the perspective of chain mediation. This study aims to provide new theoretical perspectives for existing literature by exploring the chain mediated relationship between social media addiction and academic procrastination. Based on previous research results and the above analysis, this study proposes four hypotheses, and the research hypothesis model is shown in Figure 1.

## 2 Methods

### 2.1 Design

The target survey population of this study was Chinese university students. Due to the difficulties in conducting extensive offline surveys in multiple regions of China, online research methods were considered feasible for this study. We adopted the convenience sampling method through Internet platforms to widely recruit participants to participate in this study.

### 2.2 Participants

Study participants were 832 college students from 30 provinces or municipalities directly under the central government in China. After excluding invalid questionnaires, the sample comprised 825 participants, including 617 women and 208 men, in following age groups: 18–22 years (*n* = 537), 23–26 years (*n* = 266), and 27–38 years (*n* = 23). In terms of the educational level of the participants, 588 were undergraduates, 212 were master’s students, and 25 were doctoral students.

### 2.3 Process

Before starting the investigation, we obtained the approval from the ethics review committee of the first author’s institution. The survey was conducted in June 2025. The participants were apprised regarding the study. After they provided informed consent to participate in the study, they could fill out the questionnaire. Participants spent an average of 9 min 37 s completing the questionnaire. After evaluating the data of 832 participants, two questionnaires that had a response time of less than 3 min and five questionnaires with missing data or incorrect basic demographic information were excluded, leaving 825 complete questionnaires for analysis. We conducted Monte Carlo efficacy analysis (Schoemann et al., 2017) to calculate the sample size required to meet the efficacy level (power = 0.8) for the mediation effect test. At least 481 samples were needed as per calculations, which was met in this study.

### 2.4 Measures

Social media addiction was evaluated using the Bergen Social Media Addiction Scale (Andreassen et al., 2016), which has been validated in Chinese samples (Cui and Yip, 2024; Lin and Longobardi, 2025). The questionnaire consists of 6 items (e.g., ‘Have you ever used social media to forget your personal questions?’), rated on a 5-point Likert scale, with options ranging from 1 to 5. The higher the score, the more severe the social media addiction. In this study, the McDonald’s coefficient was 0.776, indicating good reliability. The Kaiser–Meyer–Olkin (KMO) value of the scale was 0.753, indicating high validity.

Academic procrastination was measured using the Academic Procrastination Scale (Solomon and Rothblum, 1984), which has been validated in Chinese samples (Liu et al., 2023; Tian et al., 2024). The questionnaire includes 18 items (e.g., ‘Did you procrastinate while preparing for the exam.’), rated on a 5-point Likert scale, with options ranging from 1 to 5, and higher scores indicating more severe academic procrastination. In this study, Cronbach’s alpha coefficient was 0.856 and McDonald’s alpha coefficient was 0.883, reflecting the scale’s high reliability. The KMO value of the scale was 0.805, indicating high validity.

Lack of self-control was evaluated using a scale developed by Morean et al. (2014), which has been validated in Chinese samples (Ma et al., 2022; Yu et al., 2022). This scale contains seven items (e.g., ‘I can resist temptation very well.’) on a 5-point Likert scale, with four items scored positively and three items scored negatively. The higher the score, the more severe the lack of self-control. In this study, Cronbach’s alpha coefficient was 0.863 and McDonald’s alpha coefficient was 0.896, indicating good reliability. The KMO value of the scale was 0.886, indicating high validity.

The FOMO was evaluated using a scale developed by Przybylski et al. (2013), which has been validated in Chinese samples (Cheng and Jiang, 2024; Zhang et al., 2024). This scale includes 10 items (e.g., ‘I’m worried that other people’s experiences are more meaningful than mine.’), rated on a 5-point Likert scale, with scores ranging from 1 to 5. The higher the score, the more severe the FOMO. In this study, Cronbach’s alpha coefficient was 0.845, while McDonald’s alpha coefficient was 0.878, indicating good reliability. The KMO value of the scale was 0.863, indicating high validity.

### 2.5 Data analysis

We calculated the means (Ms) and standard deviations (SDs) of the scores for social media addiction, academic procrastination, lack of self-control, and FOMO in the participants. We also analyzed the correlations among these four constructs. Before conducting linear regression and mediation effect calculations, it is necessary to analyze the normal distribution of variables. Since the sample size > 300, kurtosis and skewness values were used to evaluate the normal distribution of the four variables. Mediation effects were calculated using the IBM SPSS software and Hayes’ Process Macro Model 4 (Hayes, 2017). The Bootstrap method with 5,000 repetitions was used to analyze the mediating effect and determine whether there is a mediating effect when the 95% confidence interval (CI) does not include zero. In the specific path effect analysis of the conditional process model, there is little difference in substantive conclusions obtained from the PROCESS macro and structural model methods (Hayes et al., 2017). When testing the chain mediation effect, in order to avoid increasing the complexity of the model, we chose the simple and intuitive regression analysis and the bootstrap resampling method provided by the PROCESS macro. The PROCESS macro has advantages in conducting mediation analysis (Sarstedt et al., 2020) and has been widely used in similar studies (Xu and Yan, 2023; Cao et al., 2024; Ni et al., 2025). Although structural equation modeling can evaluate model fit indices and analyze complex relationships, this study focuses on testing mediation effects along clearly specified paths rather than optimizing the overall model. Therefore, choosing this method is reasonable.

## 3 Results and analysis

### 3.1 Common methods for deviation testing

To test the common method bias in this study, we used the Harman single factor test method (Podsakoff et al., 2003). Through non rotated principal component analysis of all the projected data for the four variables of social media addiction, academic procrastination, lack of self-control, and FOMO, the results showed nine principal components with eigenvalues greater than 1, with the explanatory power of the first component as 23.641%, which is less than the critical value of 40%, indicating no common method bias.

### 3.2 Descriptive statistics and correlation analysis

The multivariate normality of the four variables, namely social media addiction, academic procrastination, lack of self-control, and FOMO, was analyzed, as shown in Table 1. Since the absolute values of skewness and kurtosis of these four variables in this study were less than 1, it can be considered that they meet the requirements of a normal distribution. The correlation results among the four variables are presented in Table 2. The research results revealed that social media addiction was associated with academic procrastination (*r* = 0.488, *p* < 0.01), FOMO (*r* = 0.475, *p* < 0.01), and lack of self-control (*r* = 0.505, *p* < 0.01). Academic procrastination was positively correlated with FOMO (*r* = 0.397, *p* < 0.01) and with lack of self-control (*r* = 0.499, *p* < 0.01). There was a positive correlation between FOMO and lack of self-control (*r* = 0.340, *p* < 0.01).

**Table 1:** Distribution of normality of the four variables.

Variable	*N*	Skewness	Kurtosis
Social media addiction	825	−0.506	0.085
Academic procrastination	825	−0.477	0.354
Lack of self-control	825	−0.423	−0.399
Fear of missing out	825	−0.218	−0.57

**Table 2:** Correlation analysis of the four variables.

Variable	Mean	Standard deviation	Social media addiction	Academic procrastination	Lack of self-control	Fear of missing out
Social media addiction	21.153	3.824	1			
Academic procrastination	58.01	11.209	0.488**	1		
Lack of self-control	22.429	5.368	0.505**	0.499**	1	
Fear of missing out	32.376	7.498	0.475**	0.397**	0.340**	1

**p < 0.01.

### 3.3 Mediating effect test

**Figure 2:**
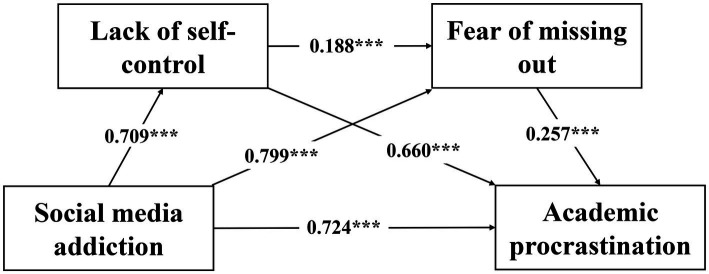
Intermediary model path coefficient diagram. ***p < 0.001.

Regression analysis revealed that social media addiction positively predicted lack of self-control (*B* = 0.709, *β* = 0.505, *t* = 16.775, *p* < 0.001) as well as FOMO (*B* = 0.799, *β* = 0.407, *t* = 11.559, *p* < 0.001), and lack of self-control positively predicted FOMO (*B* = 0.188, *β* = 0.135, *t* = 3.82, *p* < 0.001) in college students. In addition, regression analysis showed that social media addiction (*B* = 0.724, *β* = 0.247, *t* = 7.013, *p* < 0.001), lack of self-control (*B* = 0.660, *β* = 0.316, *t* = 9.590, *p* < 0.001), and FOMO (*B* = 0.257, *β* = 0.172, *t* = 5.304, *p* < 0.001) significantly predicted academic procrastination, explaining 34.7% of the variance in academic procrastination (*F* = 145.136, *p* < 0.001). According to the results of linear regression, the D-W values were all approximately 2, and the variance inflation factor (VIF) values were all less than 5, indicating that there was no multicollinearity and autocorrelation in the linear regression equation, which supports the effectiveness of the linear regression prediction model (see Table 3). A mediation model was generated based on the path coefficients (see Figure 2).

**Table 3:** Regression analysis results of the mediation model.

Dependent variable	Independent variable	*B*	*β*	*t*	*p*	95% Confidence Interval	VIF	R2	*F*	D-W
Lack of self-control							1	0.26	281.42	1.973
	Social media addiction	0.709	0.505	16.78	*p* < 0.001	[0.626, 0.792]				
Fear of missing out								0.24	129.34	1.871
	Social media addiction	0.799	0.407	11.56	*p* < 0.001	[0.663, 0.934]	1.3			
	Lack of self-control	0.188	0.135	3.82	*p* < 0.001	[0.091, 0.285]	1.3			
Academic procrastination								0.35	145.14	1.939
	Social media addiction	0.724	0.247	7.013	*p* < 0.001	[0.522, 0.927]	1.6			
	Lack of self-control	0.66	0.316	9.59	*p* < 0.001	[0.525,0.795]	1.4			
	Fear of missing out	0.257	0.172	5.304	*p* < 0.001	[0.162,0.351]	1.3			

VIF, variation inflation factor.

We calculated the path effect and its 95% CI of the chain mediation model, as shown in Table 4. The total effect of social media addiction on academic procrastination was 1.431, with direct and indirect effects of 0.724 and 0.707, respectively, accounting for 50.61 and 49.39% of the total effect, respectively. The 95% CI for both the effects did not include zero, indicating a significant impact of social media addiction on academic procrastination. Three mediating pathways were identified between social media addiction and academic procrastination. When lack of self-control and FOMO were used as separate mediators, their effect sizes were 0.468 and 0.205, respectively, accounting for 32.69 and 20.83% of the total mediating effect, respectively. When lack of self-control and FOMO were combined as chain mediators, the effect size was 0.0342. This chain mediated pathway is significantly lower than the other two independent mediated pathways. The 95% CI of the three mediation pathways was not zero, indicating significant mediation effects (see Table 4).

**Table 4:** Analysis of the results of mediating effects.

Pathway	Effect size	BootSE	BootLLCI	BootULCI	Relative mediating effect
Total effect	1.431	0.089	1.256	1.606	100.00%
Direct effect	0.724	0.103	0.522	0.927	50.61%
Indirect effect total	0.707	0.076	0.561	0.862	49.39%
Ind1: Social media addiction → Lack of self-control→ Academic procrastination	0.468	0.059	0.357	0.586	32.69%
Ind2: Social media addiction → Fear of missing out → Academic procrastination	0.205	0.045	0.123	0.298	20.83%
Ind3: Social media addiction → Lack of self-control → Fear of missing out → Academic procrastination	0.034	0.012	0.014	0.062	2.39%
Ind1 minus Ind2	0.263	0.075	0.118	0.413	/
Ind1 minus Ind3	0.434	0.061	0.317	0.556	/
Ind2 minus Ind3	0.171	0.043	0.096	0.263	/

In order to analyze whether there are gender differences in the mediating pathways between social media addiction and academic procrastination, the Bootstrap method was used to test the group differences between male and female college students (see Table 5). The confidence intervals of the three indirect mediating pathways for males and females do not include 0, indicating that each indirect mediating pathway is valid. These results indicate that social media addiction can affect academic procrastination in both male and female college student populations through lack of self-control and FOMO, respectively, and that lack of self-control and FOMO can jointly form a chain mediation between social media and academic procrastination.

**Table 5:** Gender differences in mediating effects.

Mediating pathway	Male			Female		
	Effect	BootLLCI	BootULCI	Effect	BootLLCI	BootULCI
Total effect	1.618	1.308	1.929	1.37	1.158	1.582
Direct effect	0.967	0.575	1.359	0.638	0.401	0.874
Indirect effect	0.652	0.355	0.964	0.733	0.563	0.907
Ind1: Social media addiction → Lack of self-control→ Academic procrastination	0.277	0.100	0.464	0.527	0.395	0.670
Ind2: Social media addiction → Fear of missing out → Academic procrastination	0.294	0.091	0.509	0.183	0.092	0.282
Ind3: Social media addiction → Lack of self-control → Fear of missing out → Academic procrastination	0.080	0.021	0.161	0.022	0.002	0.049

## 4 Discussion

The results of this study indicate that social media addiction can positively predict academic procrastination in college students. Lack of self-control and FOMO played mediating roles between social media addiction and academic procrastination, and together formed a chain mediation between them. This study proposed four hypotheses and confirmed all of them through mediation effect testing, providing new information on how social media addiction affects the mechanism of academic procrastination among college students. The theoretical contribution of this study is to reveal the mechanism by which social media addiction can affect academic procrastination through a chain mediated model of lack of self-control and FOMO, and this mechanism exists in both male and female college student populations, providing a new theoretical perspective for understanding the relationship between social media addiction and academic procrastination.

### 4.1 The impact of college students’ social media addiction on academic procrastination

The research results indicate that college students’ social media addiction predicted academic procrastination, thus verifying H1. This is consistent with previous research findings (Ho et al., 2024; Naushad et al., 2025). This study found that social media addiction was associated with academic procrastination, further supporting the conclusion that social media addiction can affect academic performance (Hamann et al., 2024). In addition to social media addiction, other addiction issues, such as addiction related to mobile phone dependence and excessive gaming, have also been found to be potentially associated with academic procrastination (Sujadi and Sulistiyo, 2025), supporting the view that non-material addiction can be associated with academic procrastination among college students.

Social media addiction may be associated with academic procrastination among college students in multiple ways. First, college students who spend a lot of time on social media consume a significant amount of their psychological resources and are more likely to feel tired or bored (Luo et al., 2024). This may be associated with a lack of energy and greater pressure to complete academic tasks (Zhao, 2023), which, in turn affect their progress in achieving course objectives. When college students use social media excessively, they are more likely to get accustomed to instant gratification and are unwilling to rest on time, which is associated with poor sleep quality (Berdida, 2025), which affects their academic performance. Second, a key factor contributing to academic procrastination among college students is their failure to complete the corresponding learning tasks on schedule. College students may crave feedback from others and become addicted to social media (Diefenbach and Anders, 2022), spending too much time on social media (Giunchiglia et al., 2018) and facing greater challenges in managing their time (Polites et al., 2018), making it difficult for them to adhere to academic timelines. Finally, social media addiction is an easy distraction (Cao and Tian, 2022), exposing them to negative information (Balpande et al., 2024), enhancing negative emotions that may interfere with their learning state, and affecting their ability to complete academic tasks on time.

### 4.2 The mediating role of lack of self-control between social media addiction and academic procrastination among college students

This study found that lack of self-control mediated the relationship between social media addiction and academic procrastination, thus verifying H2 and supporting previous research findings (Üztemur and Dinc, 2023). This finding is similar to the findings of a recent study on Iranian university students using structural equation modeling (Rasouli et al., 2025), providing support for the cross-cultural promotion of the conclusion that self-control mediates the relationship between social media addiction and academic procrastination. This also means that this mediating pathway has strong explanatory power in different cultural environments, reflecting the important mediating role of self-control between social media addiction and academic procrastination. In addition, previous studies have also found that other forms of digital addiction, such as smartphone addiction, can have an impact on academic procrastination through self-control (Zhao et al., 2025), which can provide new empirical data for research in this area.

Social media addiction to some extent weakens self-awareness (Dumitrescu et al., 2023) and has a negative impact on self-control, which in turn affects students’ academic performance (Ning and Inan, 2024). The self-control power model suggests that the consumption of limited self-control resources can affect an individual’s regulation of subsequent actions (Baumeister et al., 2007). Long-term excessive use of social media undoubtedly consumes a large amount of self-control resources, which can easily deplete the psychological resources necessary for individuals to regulate normal learning and daily behaviors, thereby be reducing their ability to perform normal learning tasks. Students with social media addiction may be more impulsive (Cudo et al., 2020) and have difficulty regulating and controlling their own behavior (Zahrai et al., 2022). The theory of ego depletion suggests that an individual’s willpower and psychological resources can affect their regulatory ability (Baumeister et al., 2024). When college students suffer from social media addiction and significantly deplete their self-control resources, they are more likely to experience a loss of behavioral control. Lack of self-control may have an impact on academic procrastination (Gökalp et al., 2023). The self-regulation model suggests that self-monitoring ability plays a key role in executing specific tasks and achieving expected goals (Zhao et al., 2021). For college students, many course tasks require a long time commitment, and inevitably lead to challenges and difficulties, requiring sufficient self-control to overcome them (Zhu et al., 2016). College students who lack self-control have greater emotional fluctuations and are more likely to experience negative emotions, which can easily lead to academic burnout and exacerbate academic procrastination. For them, the negative impact of social media addiction on academic procrastination may be severe, as they lack sufficient self-regulation when faced with temptations related to social media (Rashi et al., 2021; Filip et al., 2025), and face difficulties in overcoming distractions and engaging in learning. Thus, social media addiction may impact academic procrastination through lack of self-control.

### 4.3 The mediating role of FOMO on social media addiction and academic procrastination among college students

This study found that FOMO plays a mediating role between social media addiction and academic procrastination, which validates H3 and supports previous research (Wang et al., 2019; Kargin et al., 2020; Sultan, 2021; Wei and Yu, 2024). The FOMO theory suggests that people crave to maintain social connections with others, and those who heavily rely on social networks to construct themselves are more likely to be influenced (Dogan, 2019). Social media addiction is associated with college students with high social needs and unstable emotions overly focused on and dependent on social media information, making them more prone to social anxiety. Social media addiction typically means excessive use of social media, and college students may be exposed to more information about their friends’ social activities or experiences, causing concerns about being absent from important group social activities (Rifkin et al., 2025), requiring them to spend more time paying attention to social network information (Kargin et al., 2020) and making them more prone to FOMO (Elhai et al., 2025). Students with social media addiction have less activity in the real world (Sari et al., 2025), making it difficult for them to effectively regulate their psychological state through activities or exercise, which exacerbates their anxiety and FOMO on social activities with friends. This fear may affect positive emotions and learning enthusiasm, may be associated with academic procrastination. It may also be associated with physical and mental exhaustion, affecting their academic performance (Milyavskaya et al., 2018). In addition, college students with higher levels of FOMO may increase their frequency of social media use and be more likely to be distracted in their studies (Shane-Simpson and Bakken, 2024), thereby being associated with an increased risk of negative learning and academic procrastination. The negative emotional experience caused by FOMO affects the mental health of college students (Metin-Orta, 2020; Wan et al., 2025), being associated with insomnia and physical discomfort, which disrupts their normal learning behavior, reduces their learning efficiency, and increases the likelihood of academic procrastination. Therefore, social media addiction among college students can affect academic procrastination through FOMO.

### 4.4 The chain mediating role of lack of self-control and FOMO in the relationship between social media addiction and academic procrastination

This study also found that lack of self-control and FOMO have a chain mediated effect between social media addiction and academic procrastination, confirming H4 and supporting previous research (Servidio, 2021; Gugushvili et al., 2024). Previous studies have found a correlation between lack of self-control and FOMO (Sun et al., 2022; Li et al., 2023). The theory of self-control proposes that self-control resources are limited, and when this important psychological resource is excessively consumed, it can lead to a decrease in subsequent self-control effects (Muraven et al., 2007). College students with social media addiction may need to expend a significant amount of psychological resources when attempting to control themselves from excessive use of social media, which to some extent weakens their subsequent self-control abilities. When self-control is lacking, college students may find it more difficult to control their excessive attention and anxiety toward social network information, exacerbating their social anxiety and unease (Garcia and Cooper, 2024). This negative emotion may make college students pay more attention to social dynamics, worry about missing social information being associated with FOMO (Blachnio et al., 2025). The theory of emotional regulation suggests that students’ academic achievement is influenced by their emotions and regulatory abilities (Jarrell and Lajoie, 2017). FOMO is a negative emotion for college students, which can seriously affect their psychological state and weaken their emotional regulation ability. It not only distracts college students’ energy, but may also put them under significant psychological pressure, further exacerbating their academic procrastination problem (Wang et al., 2019). In the context of social media addiction, when college students experience a lack of self-control and FOMO, they are more likely to indulge in academic procrastination (Li and Ye, 2022). The non-limited theory suggests that individuals with higher demands have better self-regulation abilities, which can reduce the occurrence of procrastination behavior (Job et al., 2015). On the contrary, college students who have lower requirements for themselves often cannot effectively control their excessive use of social media, have difficulty maintaining good self-control, cannot overcome the FOMO caused by social media, and thus cannot reduce academic procrastination. In addition, the lack of self-control and FOMO make it difficult for students to effectively handle academic pressure, and they are more likely to experience academic procrastination when facing the heavy academic burden of university. Therefore, social media addiction among college students can affect their self-control ability, which in turn affects their FOMO and ultimately has an impact on academic procrastination, forming a complete chain mediation mechanism.

According to the findings of this study, in order to more effectively intervene in the impact of social media addiction on academic procrastination, the following measures need to be taken: firstly, the excessive addiction of college students to social media should be reduced through various means. Large social media platforms can develop healthy online advocacy plans for young college students, provide appropriate reminders to young users who use social media for a long time, and guide them to arrange their usage time reasonably. Schools and parents should also pay attention to students’ use of social media, provide comprehensive support and guidance to college students addicted to social media in a timely manner, and guide students to return to a reasonable state of social media use. College students with social media addiction should be aware of the negative consequences and proactively develop rational social media usage plans. Through mindfulness training (Moqbel et al., 2024), regular physical exercise (Chen et al., 2022), cultivating interests and hobbies, and setting time limits for smart device usage, they can gradually reduce their excessive dependence on social media. Secondly, it is necessary to strengthen the training of self-control among college students. Schools can regularly conduct training and practical activities that help improve self-control, gradually enhancing self-control through systematic lectures, gamified training (Ansari et al., 2024) and professional training (Tang et al., 2022). Thirdly, effective measures should be taken to alleviate the FOMO among college students. Schools should teach students how to manage social stress effectively (Yüksel et al., 2025), strengthen social skills training (Piko et al., 2025), and enhance their ability to access social information. In addition, schools can organize a variety of extracurricular activities to increase communication and interaction opportunities for college students, thereby reducing social anxiety and concerns. Fourthly, comprehensive measures should be taken to reduce the occurrence of academic procrastination. Schools should develop systematic educational programs to reduce the risk of social media addiction among college students, increase self-control training, and alleviate the FOMO, in order to reduce the occurrence of academic procrastination among students. In addition, teachers should promptly pay attention to students’ academic challenges and difficulties, and provide necessary support and intervention, such as guiding students to accept and commit to treatment methods (Lee et al., 2025), or regularly pushing out notifications through teaching management systems (Mumcu and Çebi, 2025). College students should establish clear academic goals, develop appropriate academic plans, make full use of school and social resources, and help themselves complete academic tasks on time.

### 4.5 Limitations and future research directions

Although this study has made valuable discoveries in both theory and practice, there are still some limitations that must be considered. First, we adopted convenience sampling, which, although easy to operate, may have led to biased conclusions. Future research should use more systematic and representative sampling techniques. Second, as all of our participants were from China, it is necessary to further evaluate the effectiveness of these findings before generalizing them to other countries or different cultural backgrounds. Third, our data were based on self-reported responses from participants, and not all participants may have answered truthfully. Diverse and accurate measurement methods are needed for further research. Fourth, the proportion of women in this study was very high, which may lead to biased results. In the future, gender ratios should be set to balance this. Fifth, the weak mediating effect of lack of self-control and FOMO between social media addiction and academic procrastination suggests the existence of other mediating mechanisms, and further research is necessary to clarify these. Sixth, the self-assessment scale used in this study may have a risk of social identity bias and common method bias. Although the problem of common method bias has been preliminarily ruled out through statistical testing, future research should combine multiple types of data sources for cross validation to enhance research validity. Seventh, although cross-sectional studies are suitable for exploratory research, they cannot establish causal relationships. Subsequent research needs to test the causal relationships between variables through longitudinal or experimental studies. Eighth, the chain mediation effect found in this study is relatively weak, indicating that the universality of this effect in cross-cultural contexts needs further testing, and subsequent research should strengthen relevant verification in different cultural backgrounds.

## 5 Conclusion

This study has four important findings. First, social media addiction among college students is positively correlated with their academic procrastination. Second, their lack of self-control and FOMO, individually mediate the relationship between social media addiction and academic procrastination. Finally, lack of self-control and FOMO together form a chain mediation effect between social media addiction and academic procrastination in this population. Our findings provide empirical data on the impact of social media addiction on academic procrastination, revealing the complex mechanisms of lack of self-control and FOMO, and expanding the depth of research in this area. However, the conclusions of this study are mainly based on the Chinese college student population, and there may be limitations in generalizing the findings across different cultural backgrounds. In addition, the chain mediation effect is relatively small, and its actual effects need to be carefully considered when formulating intervention measures. The research results indicate that educators should pay attention to the issue of social media addiction among college students, strengthen self-control training, improve social psychological counseling for college students to alleviate FOMO and more effectively reduce academic procrastination. In the future, researchers can conduct more in-depth research on the mechanism between social media addiction and academic procrastination, such as exploring the roles played by other important mental health variables in this relationship, seeking more precise intervention measures to alleviate their negative impact, protect the mental health of college students, and improve their academic performance.

## Data Availability

The raw data supporting the conclusions of this article will be made available by the authors, without undue reservation.
